# A Novel Homozygous *JAK3* Mutation Leading to T-B+NK– SCID in Two Brazilian Patients

**DOI:** 10.3389/fped.2018.00230

**Published:** 2018-08-20

**Authors:** Lucila A. Barreiros, Gesmar R. S. Segundo, Anete S. Grumach, Pérsio Roxo-Júnior, Troy R. Torgerson, Hans D. Ochs, Antonio Condino-Neto

**Affiliations:** ^1^Laboratory of Human Immunology, Department of Immunology, Institute of Biomedical Sciences, University of São Paulo, São Paulo, Brazil; ^2^Department of Pediatrics, Federal University of Uberlandia Medical School, Uberlândia, Brazil; ^3^Clinical Immunology, Faculdade de Medicina ABC, Santo André, Brazil; ^4^Immunology & Allergy Chief of Division, Department of Pediatrics, Ribeirão Preto Medical School, University of São Paulo, Ribeirão Preto, Brazil; ^5^Department of Pediatrics, University of Washington School of Medicine and Seattle Children's Research Institute, Seattle, WA, United States

**Keywords:** primary immunodeficiency, severe combined immunodeficiency, SCID, *JAK3*, newborn screening

## Abstract

We report a novel homozygous *JAK3* mutation in two female Brazilian SCID infants from two unrelated kindreds. Patient 1 was referred at 2 months of age due to a family history of immunodeficiency and the appearance of a facial rash. The infant was screened for TRECs (T-cell receptor excision circles) and KRECs (kappa-deleting recombination excision circles) for the assessment of newly formed naïve T and B cells respectively, which showed undetectable TRECs and normal numbers of KRECs. Lymphocyte immunophenotyping by flow cytometry confirmed the screening results, revealing a T-B+NK– SCID. The patient underwent successful HSCT. Patient 2 was admitted to an intensive care unit at 8 months of age with severe pneumonia, BCGosis, and oral moniliasis; she also had a positive family history for SCID but newborn screening was not performed at birth. At 10 months of age she was diagnosed as a T-B+NK– SCID and underwent successful HSCT. *JAK3* sequencing revealed the same homozygous missense mutation (c.2350G>A) in both patients. This mutation affects the last nucleotide of exon 17 and it is predicted to disrupt the donor splice site. cDNA sequencing revealed skipping of exon 17 missing in both patients, confirming the predicted effect on mRNA splicing. Skipping of exon 17 leads to an out of frame deletion of 151 nucleotides, frameshift and creation of a new stop codon 60 amino acids downstream of the mutation resulting in a truncated protein which is likely nonfunctional.

## Introduction

Primary immunodeficiency disorders (PIDs) are a heterogeneous group of more than 330 genetic diseases caused by more than 320 single gene defects, that lead to increased susceptibility to infections and frequently immune dysregulation (1). Severe Combined Immunodeficiency (SCID), the most devastating of cognate immunodeficiencies, is characterized by profound defects in T lymphocyte development and function, which affects cellular and humoral immunity and function. Although SCID babies frequently appear healthy at birth, they are highly susceptible to infections and present with failure to thrive, diarrhea and chronic infections due to opportunistic pathogens. Without treatment, this condition is invariably fatal, but if recognized early, hematopoietic stem cell transplantation (HSCT) or gene therapy are curative (2).

A typical or classic SCID is defined as a patient with less than 300 autologous CD3^+^ T cells per microliter of blood. Traditionally, SCID patients can be classified according to the specific immunologic phenotype as T cell–deficient but with normal B cells (T–B+) SCID or both T cell– and B cell–deficient (T–B–) SCID, with a further subdivision depending on the presence or absence of NK cells, resulting in 4 distinct immunophenotypes (T-B+NK+, T-B+NK–, T-B-NK+, T-B-NK–). There frequently is a correlation between the immunophenotype and underlying genetic defect causing the SCID phenotype (3). While T-B– SCID phenotypes are largely due to defects in antigen receptor rearrangements, the majority of T-B+ SCIDs are related to cytokine signaling abnormalities, in particular, caused by mutations in the common-gamma chain (*IL2RG*) gene, the Janus kinase 3 (*JAK3*) gene or the IL-7 receptor alfa chain (*IL7RA*) gene, accounting for 67–74% of all SCIDs cases (4). The common-gamma chain is part of several cytokine receptors (IL-2, IL-4, IL-7, IL-9, IL-15 and IL-21) (5), which following cytokine activation interacts with the intracellular tyrosine kinase Jak-3, resulting in STAT3 phosphorylation, a transcription factor indispensable for cell growth and control of hematopoietic cell development (5). Absence of the common gamma chain or Jak-3 results in T-B+NK– SCID with clinical symptoms that are indistinguishable between X-linked SCID due to mutation in the *IL2RG*gene, and the autosomal recessive SCID due to *JAK3* mutations.

Here we present two Brazilian girls with SCID from two unrelated kindreds with the same novel homozygous mutation in *JAK3*, that results in skipping of exon 17, frameshift, predicting a truncated Jak-3 protein which is likely nonfunctional, leading to the SCID phenotype.

## Case presentation

**Figure 1:**
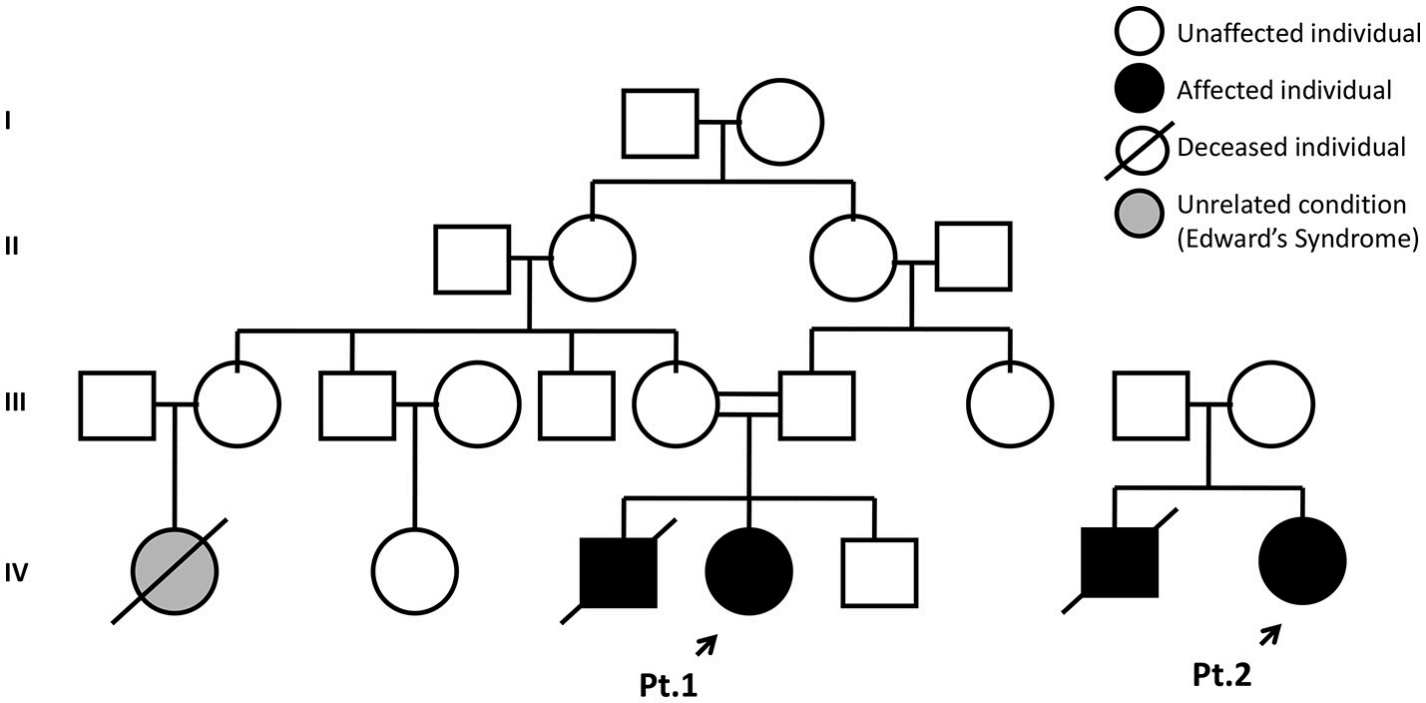
Family pedigree of Patients 1 and 2. Probands are indicated by the black arrow.

Patient 1 was born to healthy consanguineous parents (first-degree cousins), weighting 3,165 g, without neonatal complications. She was referred to our service by an immunologist due to the appearance of a dyschromic rash on her face and a positive family history for SCID Figure 1—a brother died at 6 months of age following admission to a local hospital with failure to thrive associated with chronic diarrhea and septic shock unresponsive to treatment. An immunodeficiency was suspected and intravenous immunoglobulin administered. There was a slight improvement but he developed sepsis within 7 days, leading to multiple organ failure and death before a proper diagnosis was made; *post mortem* results showed bone marrow aplasia.

Due to Patient's 1 family history, she was not vaccinated with BCG or any other live or attenuated organisms. She had her first hematological assessment at 2 months of age, which showed normal cell numbers except for a high platelet count of 683,000; immunological analysis revealed low numbers of CD3^+^, CD4^+^, and CD8^+^ T cells Table 1 for her age according to the reference range established for Brazilian population (6). The patient's T cells were low, but no completely absent, however it was no possible to assess TCR repertoire or lymphocyte memory subsets to exclude the possibility of maternal cell engraftment. One month later the patient's immunological profile was characterized by normal B cell numbers but T and NK cells were below the 10th percentile, with a pronounced fall in CD4^+^ T cells. Still, based on the family history and the low lymphocyte numbers, the diagnose of T-B+NK– SCID was entertained. The patient was constantly monitored, started on Co-trimoxazole and acyclovir prophylaxis while waiting for a matched bone marrow donor. At 4 ½ months of age, quantification of TRECs (T-cell receptor excision circles), and KRECs (kappa-deleting recombination excision circles) by quantitative PCR became available as part of a pilot program our group at University of São Paulo initiated. TREC and KREC numbers are indirect assessments of recent thymic emigrants (naïve T cells) and recent B cell emigrants from the bone marrow (naïve B cells) in newborns, respectively, and they can be quantified using dried blood spots on Guthrie cards, which are routinely used for newborn screening of certain diseases in Brazil and other countries (7). Thus, blood was collected on Guthrie cards and TREC and KREC quantification was used to assess the numbers of naïve T and B cells of Patient 1. While TRECs were undetectable, KRECs were normal (158 molecules / μL of blood) (8), in accordance with the abnormal lymphocyte's immunophenotyping, which showed a T-B+ profile. Repeated immunophenotyping at age 11 and 12 months revealed decreasing T and NK cell numbers Table 1. She was successfully transplanted at age 1 year and 3 months with 100% of chimerism and was well 2 and a half years post HSCT.

**Table 1:** Lymphocytes immunophenotyping of Patient 1 in different ages.

**Cell counts (cells/mm^3^)**	**2 m**	**3 m**	**11 m**	**12 m**
Total lymphocyte count	↓ 2212.6 (3720–8426)	↓ 2760 (3720–8426)	↓ 1820 (3720–8426)	↓ 1206.6 (3245-6981)
**CD3**	↓ 875 (2093.1–5054.5)	↓ 1529 (2093.1–5054.5)	↓ 311.2 (2093.1–5054.5)	↓ 180.9 (1906.9–4313.9)
**CD4**	↓ 260 (1360.9–3265.5)	↓ 61.2 (1360.9–3265.5)	↓ 16.1 (1360.9–3265.5)	↓ 23.5 (957.2–2727.1)
CD4^+^CD45RA^+^CCR7^+^ (naïve)	ND	ND	↓ 0 (366.2–2100.3)	↓ 0.3 (290.8–1634.8)
CD4^+^CD45RA_−_CCR7^+^ (central memory)	ND	ND	↓ 4.5 (130.4–430.4)	↓ 11.9 (123.8–433.9)
CD4^+^CD45RA^−^CCR7^−^ (peripheral memory)	ND	ND	↓ 5.1 (73.7–293.7)	↓ 8.7 (59.9–335.4)
CD4^+^CD45RA^+^CCR7^−^ (terminal differentiation)	ND	ND	↓ 0.2 (98.6–980.8)	↓ 2.4 (93.8–741.3)
**CD8**	↓ 90 (559.5–1802.5)	↓ 162.1 (559.5–1802.5)	↓ 66.2 (559.5–1802.5)	↓ 25.3 (563.3–1753.2)
CD8^+^CD45RA^+^CCR7^+^ (naïve)	ND	ND	↓ 0.3 (55.6–582.5)	↓ 0.7 (101–565.7)
CD8^+^CD45RA^−^CCR7^+^ (central memory)	ND	ND	↓ 3.8 (13.5–60.8)	↓ 1.36 (12.5–46.7)
CD8^+^CD45RA^−^CCR7^−^ (peripheral memory)	ND	ND	↓ 30.2 (51.3–652.3)	↓ 13.2 (76.2–564.8)
CD8^+^CD45RA^+^CCR7^−^ (terminal differentiation)	ND	ND	↓ 9.2 (355.2–1305.7)	↓ 9.8 (196.9–756.6)
**CD19**	ND	1145.4 (648.8–2072.9)	1072 (888.1–2720)	↓ 299.2 (648.8–2072.3)
CD19^+^CD27^−^ (naïve)	ND	ND	10.7	6.8
CD19^+^CD27^+^ (memory)	ND	ND	1061.2	290.2
**CD16**^+^**CD56**^+^	ND	↓ 38.64 (153–702.9)	↓ 47.3 (163.7–800.6)	↓ 13.2 (153–702.9)

*Reference ranges for the Brazilian population (6) in parenthesis (percentile 10–90). ND, not done. The arrows indicate values that are below the 10th percentile*.

Patient 2 was born to healthy non-consanguineous parents, weighting 3,800 g and showed no signs of an immunodeficiency at birth. This female patient had a previous suspicion of a primary immunodeficiency in her family, she had a brother who died at 8 months of age due to severe pneumonia and a history of BGCosis, oral moniliasis, and pityriasis alba, but there was no time for a final diagnosis.

Pt.2 was admitted at 8 months of age to an intensive care unit (ICU) for the first time due to severe pneumonia. Two months later she was hospitalized again due to a pneumonia caused by *Pneumocystis jirovecii*. While in the ICU she was also found to have BCGosis, developed diarrhea associated with *Klebsiella* and oral moniliasis. Lymphocyte subset analysis revealed total absence of CD3^+^ T cells, with B cells being 644 cells/μL and NK(CD3^−^CD16^+^CD56^+^) being 161 cells/μL, both below the 10th percentile for her age; additional immunoglobulin measurements showed virtually no Igs (IgG: 27 mg / dl; IgA: 2 mg/ dl; IgM: 3.8 mg/dl). Based on these results, Pt.2 was characterized as a T-B+NK+ SCID and was referred to HSCT and underwent haploidentical HSCT from a family donor (matched brother). Chimerism was approximately 90%. Following the procedure, Pt.2 developed 2 episodes of pneumonia of unknown etiology and pulmonary tuberculosis; cutaneous lesions in the scalp, nose and trunk; adenomegaly (fungal infection) and oral and genital moniliasis, with prompt response to antibiotics and antifungals. Six months after HSCT, T cell numbers and immunoglobulin levels are raising (CD3^+^CD4^+^: 646 cells/μL; CD3^+^CD8^+^: 551 cells/μL; CD19: 456 cells/μL; CD3^−^CD16^+^CD56^+^: 209 cells/μL; IgG: 770 mg/dl; IgA: 42 mg/dl; IgM: 61 mg/dl) and the infections are under control, but she is still being followed by a pneumologist due to respiratory problems caused by the previous infections.

**Figure 2:**
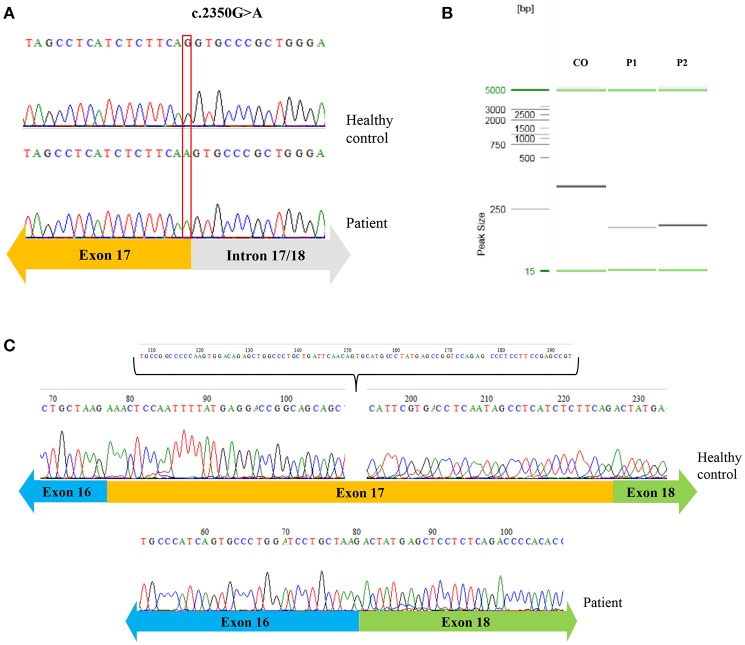
Novel homozygous mutation on JAK3. Sequence analysis of the patients compared to a healthy control showing in the genomic DNA the guanine [G] per adenosine [A] single nucleotide substitution **(A)**; agarose gel electrophoresis of Pt.1 and Pt.2 compared to a control, showing fragments with a smaller molecular weight in the patients **(B)**; sequencing of cDNA of patient 1 revealing the absence of the exon 17 in the patient **(C)**.

We then sought to investigate the genetic defect underlying the SCID phenotype of these patients, using DNA extracted from peripheral blood mononuclear cells (PBMCs). Exome sequencing was performed for Patient 1, revealing a homozygous missense mutation in the *JAK3* gene, c.2350G>A, which was subsequently confirmed by Sanger sequencing Figure 2A. Pt.2 also presented the same mutation in *JAK3*. This mutation is present in heterozygosity in gnomAD (genome aggregation database) in one African e one Latino individual (rs760051760), but it was not found in its homozygotic form in any public mutation databases searched, such as OMIM (ClinVar), HGMD, and UCSC genome browser. This mutation is predicted to cause an amino acid substitution at the 784th residue (D784N) of the Jak-3 protein, which is a part of the pseudo-kinase domain, responsible for regulating the kinase activity of Jak-3 (9). This region is highly conserved and *in silico* analysis with different tools (PolyPhen2, SIFT and Mutation Taster) predicted that the c.2350G>A mutation is potentially pathogenic and could affect the splice site (10). To investigate this possibility, we isolated mRNA from the patient's PBMCs and reverse transcribed it into cDNA. After amplifying a fragment containing exons 16 to 18 of *JAK3* by polymerase chain reaction (PCR), agarose gel electrophoresis revealed that both patients had fragments with smaller molecular weight than the fragment of a control individual Figure 2B, consistent with a splicing defect. Finally, Sanger sequencing showed an absence of the entire exon 17 when compared to a healthy control sequence Figure 2C, confirming the prediction that the proximity of the mutation to the donor splice site disrupts the splicing process.

## Discussion

In the present work we present 2 Brazilian patients form 2 non-related kindreds with the same novel homozygous mutation in *JAK3*, c.2350G>A, leading to SCID. Patient 1 was diagnosed with SCID before symptoms appeared due to a positive family history and the availability of TREC and KR EC quantification for the assessment of naïve T and B cells. The clearance of infections at the time of bone marrow transplantation contributed to the excellent outcome (11). Patient 2 presented at age 8 months with symptoms suggesting SCID and even though this patient also had a positive family history for SCID, there was no previous investigation for the disease. She underwent successful HSCT and is currently doing well, despite the initial complications of active infections at the time of the procedure. Because both patients had the same mutation and were originally from the same state, the issue of consanguinity was raised, but the parents were not aware of any consanguinity.

Mutations in the exon-intron junction are responsible for about 10% of pathogenic mutations and among those, 91% of splicing mutations occur in the last 5 nucleotides of the exon and the first 15 nucleotides of the intron suggesting that variants affecting the donor splicing region are more frequently pathogenic than variants in the acceptor region (10). *In silico* analysis of the mutation we identified in 2 infant girls indicated that the nucleotide exchange could impair splicing, and cDNA analysis confirmed the skipping of exon 17.

Jak-3 is a non-receptor tyrosine-kinase expressed predominantly in immune cells that associates with the common-gamma chain (γc) subunit of various cytokine receptors (IL-2, IL-4, IL-7, IL-9, IL-15, IL-21) and upon stimulation activates other kinases and transcription complexes (STATs) (12). Jak-3 is a member of the Janus kinases family of proteins, all of which share 7 conserved regions called Janus homology (JH) domains (13). Because exons 17 to 23 encode the JH1 domain, the catalytic subunit of the Jak-3 protein (9), and this region in highly conserved among different species, it is probable that the absence of the first exon of this conserved portion of a catalytic domain could be damaging by itself. Furthermore, skipping of exon 17 results in frameshift beginning in exon 18, and the creation of a stop codon 60 amino acids downstream of the splice-site mutation (p.D784Nfs^*^60), which likely abrogates catalytic activity and if the protein is expressed, it is probably inactive. Moreover, Walshe et al. (14) described a male *JAK3* deficient SCID patient with an adjacent mutation (c.2350+1G>T), also affecting splicing, in which STAT5 phosphorylation was completely absent. Thus, the mutation c.2350G>A found predicts a truncated protein which is likely to be nonfunctional, but due to the initial numbers of T cells in Patient 1, it is unclear whether there could be any residual activity of Jak-3 or if the cell numbers were a result of a maternal engraftment.

Without newborn screening, SCID is rarely diagnosed until the patient develops recurrent infections (15), with the exception of those newborns from families that have a history of deceased individuals who died of infections, raising the possibility of PID. This scenario has changed since the introduction of newborn screening for SCID in most states of the U.S. using TRECs (16). Implementation of newborn screening increased the incidence of SCID from 1:1,000,000 to 1: 58,000 (17) in the U.S., indicating that in the absence of newborn screening, a large proportion of SCID patients die without a diagnosis. With newborn screening in place, early diagnosis and treatment are possible before symptoms appear. Newborn screening for SCID is a reliable, cost-effective and feasible method to reach this goal (18, 19). Recently, two articles were published reporting the results of two Brazilian pilot programs on newborn screening using TRECs (20) and then TRECs and KRECs (8), demonstrating that the technology and know-how to implement newborn screening is available in Brazil.

## Concluding remarks

This work revealed a novel splice site mutation in the *JAK3* gene and the methodologies we used to access the functional consequences of this variation. The c.2350G>A mutation at the end of exon 17 of *JAK3* causes skipping of exon 17 and creates a premature stop codon 60 amino acids downstream of the mutation predicted to annihilate the catalytic activity of the Jak-3 protein, leading to T-B+NK– SCID in the 2 female patients from two unrelated families. Despite the fact that both patients carried the same mutation and had positive family histories, one girl was diagnosed and treated before the appearance of symptoms while the other girl was not diagnosed until the appearance of life threatening infections. This discrepancy between these 2 cases illustrates the relevance of early diagnosis and curative treatment for SCID patients, which correlates with better outcomes. Newborn screening for SCID is a reliable, cost-effective and feasible method to reach this goal and is currently being used in several countries.

## Ethics statement

The work was conducted in accordance with the Human Research Ethics Committee of the Biomedical Sciences Institute of University of São Paulo (CAAE 36364214.8.0000.5467) and written and informed parental consent was obtained for publication of this case report.

## Author contributions

AC-N conceived the study. LB conceived and wrote the first draft of the case report and created the table and figures. PR-J and AG were involved in the clinical care of the patients. LB and GS performed the experimental procedures that lead to the case report. HO, TT, and AC-N were involved in concept development and editing of the manuscript. All authors reviewed and revised the manuscript and approved the final submitted version.

### Conflict of interest statement

The authors declare that the research was conducted in the absence of any commercial or financial relationships that could be construed as a potential conflict of interest regarding this publication.
