# Corrigendum: A Novel Homozygous *JAK3* Mutation Leading to T-B+NK- SCID in Two Brazilian Patients

**DOI:** 10.3389/fped.2018.00358

**Published:** 2018-11-23

**Authors:** Lucila A. Barreiros, Gesmar R. S. Segundo, Anete S. Grumach, Pérsio Roxo-Júnior, Troy R. Torgerson, Hans D. Ochs, Antonio Condino-Neto

**Affiliations:** ^1^Laboratory of Human Immunology, Department of Immunology, Institute of Biomedical Sciences, University of São Paulo, São Paulo, Brazil; ^2^Department of Pediatrics, Federal University of Uberlandia Medical School, Uberlândia, Brazil; ^3^Clinical Immunology, Faculdade de Medicina ABC, Santo Andre, Brazil; ^4^Immunology & Allergy Chief of Division, Department of Pediatrics, Ribeirão Preto Medical School, University of São Paulo, Ribeirão Preto, Brazil; ^5^Department of Pediatrics, University of Washington School of Medicine and Seattle Children's Research Institute, >Seattle, WA, United States

**Keywords:** primary immunodeficiency, severe combined immunodeficiency, SCID, *JAK3*, newborn screening

Pérsio Roxo-Júnior was not included as an author in the published article. The authors apologize for this error and state that this does not change the scientific conclusions of the article in any way. The original article has been updated.

## Author contributions

AC-N conceived the study. LB conceived and wrote the first draft of the case report and created the table and figures. PR-J and AG were involved in the clinical care of the patients. LB and GS performed the experimental procedures that lead to the case report. HO, TT, and AC-N were involved in concept development and editing of the manuscript. All authors reviewed and revised the manuscript and approved the final submitted version.

### Conflict of interest statement

The authors declare that the research was conducted in the absence of any commercial or financial relationships that could be construed as a potential conflict of interest.

